# Small Bowel Injury in Peritoneal Encapsulation following Penetrating Abdominal Trauma

**DOI:** 10.1155/2013/379464

**Published:** 2013-02-26

**Authors:** K. Naidoo, S. Mewa Kinoo, B. Singh

**Affiliations:** Department of Surgery, Nelson R Mandela School of Medicine, University of KwaZulu-Natal, 719 Umbilo Road, Congella 4013, South Africa

## Abstract

Small bowel encapsulation is a rare entity which is usually found incidentally at autopsy. We report the first case of peritoneal encapsulation encountered serendipitously at laparotomy undertaken for penetrating abdominal trauma and review the literature on peritoneal encapsulation. We also compare this phenomenon to abdominal cocoon and sclerosing encapsulating peritonitis.

## 1. Introduction

The terms peritoneal encapsulation (PE), abdominal cocoon, and sclerosing encapsulating peritonitis (SEP) are used interchangeably to describe the rare conditions of small bowel encapsulation. The literature on this subject is dominated by case reports. Presently there is no consensus on the classification of these 3 distinct pathological entities that effectively constitute small bowel encapsulation [1]. Whereas PE is an embryological disorder and abdominal cocoon is an idiopathic condition, SEP is today increasingly associated with peritoneal dialysis as well as a variety of other conditions [2].

## 2. Case Presentation

A 40-year-old male patient presented to our surgical unit following an isolated stab wound to the abdomen in the region of the epigastrium. The patient had no medical history of note.

On examination, the patient was noted to be haemodynamically stable. Abdominal examination revealed peritonitis. The admission chest and abdominal radiographs were noted to be normal.

**Figure 1:**
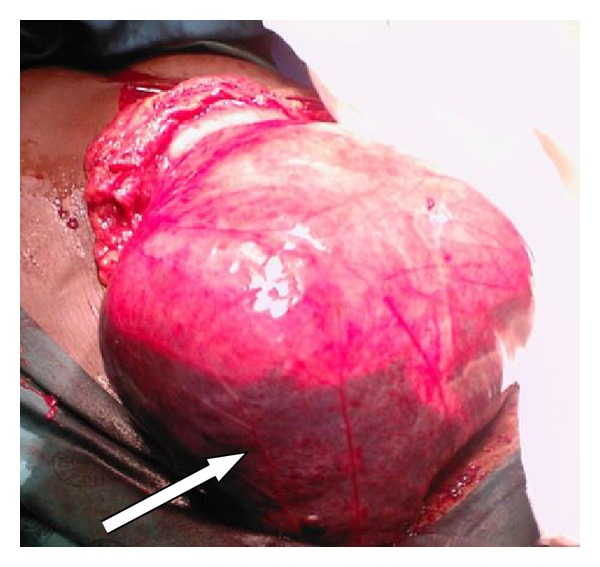
Peritoneal sac encountered at laparotomy; note free blood pooled at the bottom of the peritoneal sac (indicated by arrow).

At laparotomy the entire small bowel was encapsulated in a peritoneal sac. The peritoneal sac was noted to be attached to the ascending and descending colon laterally, to the transverse colon superiorly, and to the pelvic peritoneum inferiorly. The peritoneal sac was transparent and was noted to contain blood. The sac was not attached to the underlying small bowel and neither to the abdominal wall parietes nor to the greater omentum (Figure 1).

**Figure 2:**
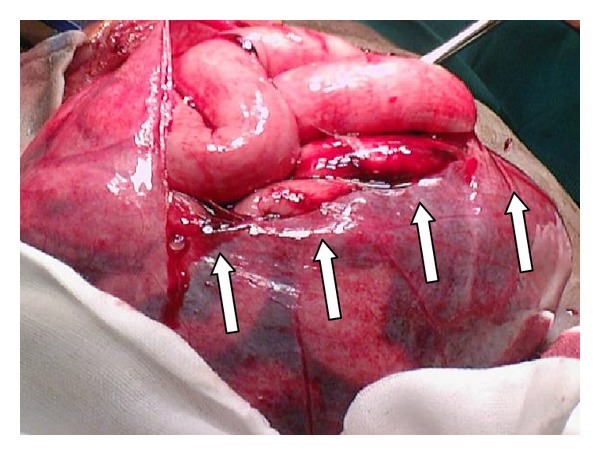
Multiple small bowel perforations (indicated by arrows).

A perforation in the peritoneal sac was noted at the site of the stab. On opening the peritoneal sac the small bowel was noted to be freely mobile. Multiple small bowel perforations were evident, probably due to the concertina effect of the bowel layered within the peritoneal sac (Figure 2). 

**Figure 3:**
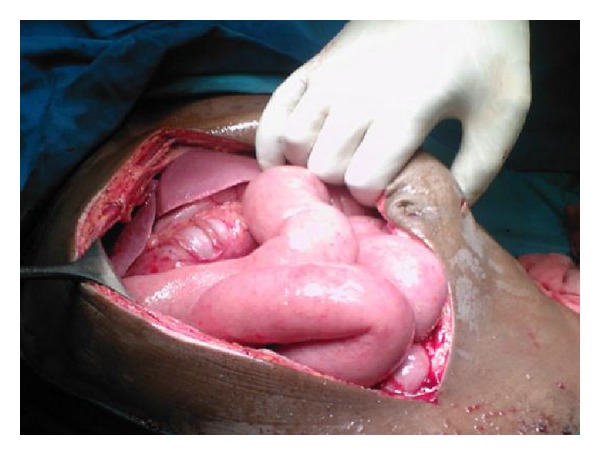
On opening the peritoneal sac the small bowel was noted to be free of adhesions and fully mobile.

The sac was excised after the repair of the small bowel perforations (Figure 3). The patient made an eventual recovery and was discharged on the 5th postoperative day.

## 3. Discussion

PE is a congenital abnormality in which the small bowel is contained in an accessory peritoneal sac derived from the yolk sac. This condition is considered to develop in the 12th embryological week following the abnormal return of the physiological umbilical hernia containing the midgut into the abdominal cavity. The accessory peritoneal sac is attached laterally to the ascending and descending colon, superiorly to the transverse colon, and to the parietal peritoneum inferiorly. A segment or entire small bowel extending from the duodenojejunal flexure to the ileocaecal junction (as in the case presented) may be contained in the accessory peritoneal sac. As evident in this paper, the greater omentum covers the sac but is not attached to it [3].

PE was first reported by Cleland in 1868. Defining the true incidence of PE has been hampered by the failure to distinguish this condition from abdominal cocoon and SEP. The literature suggests that incidence of PE ranges between 20 and 40 cases [4–6]. PE is usually an incidental finding noted at autopsy or at laparotomy, as in this paper [7]. Rarely, PE may present with either complete or incomplete small bowel obstruction in patients who usually have a long history of abdominal pain [8, 9]. Small bowel gangrene and aortic occlusion have each been reported once [10, 11].

The literature supports the excision of the peritoneal sac when encountered incidentally at laparotomy with lysis of interloop adhesions, if present, in symptomatic patients. Histological examination of the excised peritoneal sac invariably demonstrates normal peritoneum without signs of inflammation [12]. 

PE must be differentiated from SEP and the abdominal cocoon phenomenon. These are distinctly different entities. 

SEP was first described by Owtschinnkow in 1907 as “peritonitis chronic fibrosa incapsulata.” SEP is an acquired condition characterized by the covering of the small bowel with a thick grayish white fibr collagenous membrane. SEP is associated with chronic ambulatory peritoneal dialysis, the beta-blocker protocol (now withdrawn from use), recurrent peritonitis, ventriculoperitoeal and peritoneovenous shunts, sarcoidosis, tuberculosis, Mediterranean fever, protein-S deficiency, following liver transplantation, systemic lupus erythematosus, and fibrogenic foreign material [13].

The abdominal cocoon was first described by Foo et al. in 1978 [14]. Classically, this condition was described as occurring in young adolescent females from the tropical and subtropical countries. However, case reports from temperate zones have been reported in all age groups regardless of gender [15–17].

The etiology of the abdominal cocoon is poorly understood. Various theories have been proffered, including retrograde menstruation with a superimposed viral infection, retrograde peritonitis, and cell-mediated immunological tissue damage incited by gynecological infection.

It is probable that the abdominal cocoon is the result of “subclinical” peritonitis. The abdominal cocoon has been described as “idiopathic SEP”. The small bowel is encapsulated by a fibrocollagenous membrane in a manner not dissimilar to that encountered in SEP. The association with embryologic abnormalities such as greater omentum hypoplasia and mesenteric vessel malformation suggests that developmental abnormality may be a probable etiology [18]. 

Notwithstanding the reported differentiation of SEP and abdominal cocoon on the basis of etiology, it is reasonable to assume that these conditions belong to a similar pathological process resulting in the fibrous encapsulation of the small bowel.

In patients presenting with small bowel obstruction associated with the fibrous encapsulation of the small bowel, 2 clinical signs have been described. The first is a fixed, asymmetrical distension of the abdomen, which does not vary with peristaltic activity due to the unvarying position of the fibrous capsule. The second is the difference in the consistency of the abdominal wall to palpation. The bowel proximal to the capsule can distend and is soft to palpation, as opposed to the flat area that is firm, due to the dense fibrous capsule that encases the underlying small bowel [19]. 

Although standard radiographic studies are usually normal, it has been suggested that a combination of barium meal will follow through studies, and abdominal computed tomography may contribute to making a preoperative diagnosis. In abdominal cocoon, barium studies may demonstrate a serpentine-or concertina-like configuration of dilated small bowel loops in a fixed U-shaped cluster and delayed transit of the contrast medium [20]. 

Computed tomography of the abdomen may demonstrate congregation of small bowel loops to the center of the abdomen encased by a soft-tissue density mantle representing the peritoneal membrane; other features include signs of obstruction, fixation of intestinal loops, bowel wall thickening, ascites, and localized fluid collections [21–23]. 

Despite anecdotal reports of a preoperative diagnosis of peritoneal encapsulation being established, in the majority of cases this is fortuitous particularly in the absence of discerning clinical signs. However, a better awareness of this condition with appropriate use of imaging techniques may facilitate preoperative diagnosis [18].
